# Comparison of a New Gold Immunochromatographic Assay for the Rapid Diagnosis of the Novel Influenza A (H7N9) Virus with Cell Culture and a Real-Time Reverse-Transcription PCR Assay

**DOI:** 10.1155/2014/425051

**Published:** 2014-04-14

**Authors:** Changzhong Jin, Nanping Wu, Xiaorong Peng, Hangping Yao, Xiangyun Lu, Yu Chen, Haibo Wu, Tiansheng Xie, Linfang Cheng, Fumin Liu, Keren Kang, Shixing Tang, Lanjuan Li

**Affiliations:** ^1^State Key Laboratory for Diagnosis and Treatment of Infectious Diseases, The First Affiliated Hospital, School of Medicine, Zhejiang University, Hangzhou 310003, China; ^2^Collaborative Innovation Center for Diagnosis and Treatment of Infectious Diseases, Hangzhou 310003, China; ^3^National Engineering Laboratory of Point-of-Care Tests, Guangzhou Wondfo Biotech Co. Ltd., Guangzhou 510641, China; ^4^School of Bioscience and Bioengineering, South China University of Technology, Guangzhou 510640, China

## Abstract

We assessed a colloidal gold immunochromatographic assay (GICA) for rapid detection of influenza A (H7N9) and compared it with reverse-transcription-polymerase chain reaction (RT-PCR) and viral culture. Samples from 35 H7N9 infected patients were collected, including 45 throat swab samples, 56 sputum samples, and 39 feces samples. All samples were tested by GICA, viral culture, and RT-PCR. GICA specifically reacted with recombinant HA proteins, virus lysates, and clinical samples from H7 subtype viruses. Compared with RT-PCR, GICA demonstrated low sensitivity (33.33%) but high specificity (97.56%). The positive rate of GICA tests for samples collected in the period from 8 to 21 days after contact with poultry was much higher than those for samples collected before or after this period. Compared with viral culture, GICA showed sensitivity of 91.67% and specificity of 82.03%. Sputum specimens were more likely to test positive for H7N9 virus than samples from throat swabs and feces. The GICA-based H7 test is a reliable, rapid, and convenient method for the screening and diagnosis of influenza A (H7N9) disease, especially for the sputum specimens with high viral load. It may be helpful in managing H7N9 epidemics and preliminary diagnosis in early stages in resource-limited settings.

## 1. Introduction

Since the first human case of influenza A (H7N9) virus infection was identified in China, a total of 347 infected patients were confirmed as of February 18, 2014, with a total of 109 deaths [1]. Neuraminidase inhibitors can inhibit the growth of influenza A viruses at the early stage of the disease [2, 3], and laboratory testing has demonstrated that H7N9 viruses are sensitive to neuraminidase inhibitors [4, 5]. Rapid and accurate diagnoses are critical for the treatment of patients with influenza A (H7N9) infections, as well as for the control of infections and the prevention of epidemics [6]. Cell culture and real-time reverse-transcription-polymerase chain reaction (RT-PCR) have been widely used for identifying influenza viruses in clinical settings. However, these methods are time-consuming and labor-intensive, and the requirements for equipment, specific laboratory conditions, and technical personnel are high and thus are not suitable for resource-limited regions, such as in primary care settings. Currently, reported cases of H7N9 infection are confirmed by RT-PCR or cell culture, or both [4, 7–10]. Therefore, a rapid and convenient H7N9 test is needed for early diagnosis.

The colloidal gold immunochromatographic assay (GICA) is a recently developed immunochromatographic technique for the identification of influenza A viruses with several notable advantages, such as the lack of requirement for any sample pretreatment, low sample volume requirement, ease of operation, rapid turnaround time, low cost, no cross-reactions, and no equipment requirements [11]. Recently, a new GICA for the rapid diagnosis of H7 influenza A viruses was developed by Guangzhou Wondfo Biotech Co. Ltd. Considering the advantages of GICA, it could be a potentially useful tool for the rapid diagnosis and screening of H7N9 viruses, if it was proven to be of comparable performance with other diagnostic methods. In this study, we first tested the sensitivity and specificity of the GICA for detecting recombinant influenza H7 hemagglutinin (HA), virus lysates, and clinical samples. Then we compared the results of the GICA with viral culture and the RT-PCR assay. We found that the GICA specifically reacted with recombinant HA protein, virus lysates, and clinical samples from H7 subtype viruses and was more sensitive than viral culture but less sensitive than RT-PCR. For the detection of samples with a high viral load, GICA performed similarly to the RT-PCR assay, especially with sputum samples. Our results indicated that GICA is an effective alternative method for the effective detection of H7N9 virus infections and surveillance, especially in resource-limited settings.

## 2. Materials and Methods

### 2.1. Influenza Virus Proteins

Recombinant HA proteins of H7N9 (A/Shanghai/2/2013), H7N7, H5N1, H3N2, and H1N1 were purchased from Immune Technology Corp. (NY, USA). In addition, recombinant HA proteins of H7N9 (A/Anhui/1/2013) and H7N7 were kindly provided by Guangzhou Institute of Respiratory Diseases (Guangzhou, China) while inactivated H5N9 and H9N2 virus lysates were obtained from South China University of Technology (Guangzhou, China).

### 2.2. Clinical Patients and Samples

Thirty-five H7N9 virus-infected patients were admitted to the First Affiliated Hospital, College of Medicine, Zhejiang University, from April 1 to May 17, 2013. H7N9 infections were diagnosed by clinical manifestation and confirmed by RT-PCR. Serial samples were prepared from the patients. In total, 45 throat swab samples, 56 sputum samples, and 39 fecal samples were collected and then diluted in 1 mL PBS. The diluted solution of samples was sterilized by filtration. All samples were tested by GICA, viral culture, and RT-PCR. The fresh samples were used for virus isolation in cell culture immediately after collection, and frozen and thawed samples were used for RT-PCR and GICA. To test the specificity of GICA, 66 throat swab samples and 25 sputum samples were collected from patients with different influenza A subtypes and other respiratory pathogen infections, including mycobacterium tuberculosis, mycoplasma pneumonia, H1N1 virus, and measles virus. All these controls had no contact with H7N9 infected patients and poultry, and the negative status of H7N9 infection was confirmed by RT-PCR.

All patients provided informed consent and the study was approved by the institutional review board of the First Affiliated Hospital, College of Medicine, Zhejiang University (reference number 2013-131).

### 2.3. GICA Test

**Figure 1:**
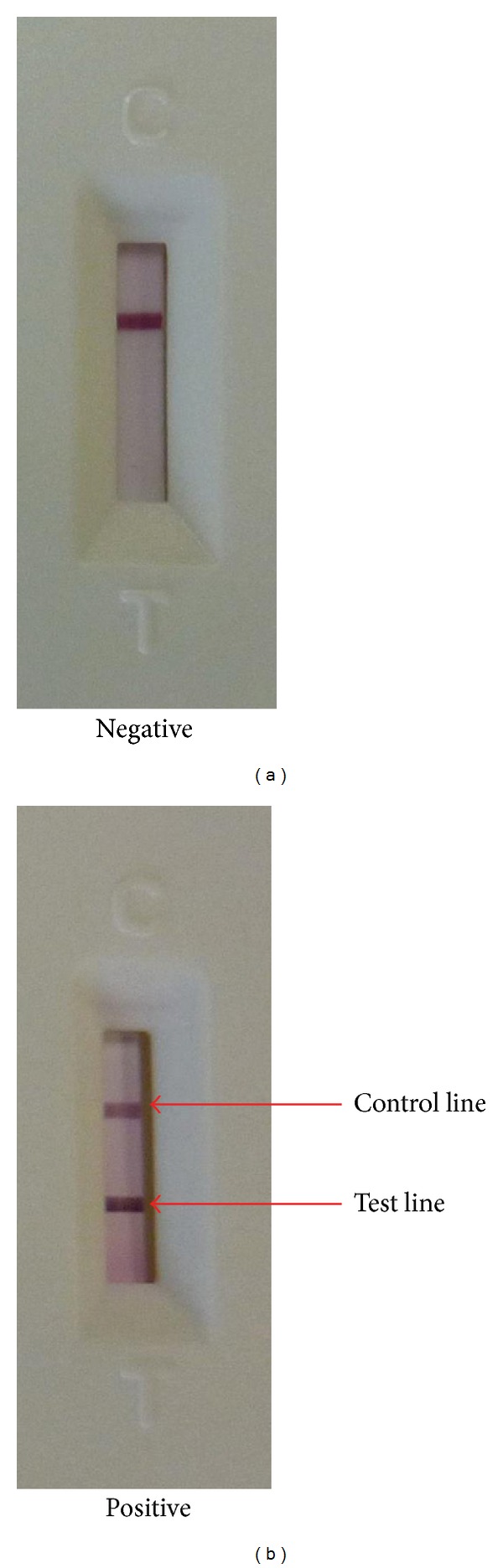
Gold immunochromatographic rapid assay of H7 virus ((a) negative; (b) positive).

A new GICA-based influenza A virus (H7 subtype) rapid test kit was developed by Guangzhou Wondfo Biotech Co. Ltd. (Guangzhou, China). This test is a sandwich-type immunoassay that includes an anti-H7-C1 monoclonal antibody and a colloidal gold-labeled anti-H7-C2 monoclonal antibody. The tests were performed according to the manufacturer's instructions. Briefly, the frozen and thawed samples were diluted in 1x dilution buffer and then were dropped onto the test card. The results were read after 15 minutes. A single red quality control line indicates a negative result; red quality control and test lines indicate a positive result (Figure 1).

### 2.4. Viral Culture

Viral culture for all samples was carried out in the Biosafety Lab (level 3) of the First Affiliated Hospital, Zhejiang University. The procedures for influenza virus culture were described before [12, 13]. Madin Darby canine kidney (MDCK) cells were cultured in minimal essential medium (MEM) with 10% fetal calf serum at 37°C in a humidified 5% CO_2_ incubator. After the monolayer cells grew approximately 80–90% confluent on the bottom of the 24-well plate, the cells were washed three times with PBS and then inoculated with 0.1 mL of diluted sample solution. After 2 h of absorption at 37°C, the plate was washed twice with PBS, then filled with 0.5 mL MEM containing antibiotic/antimycotic solution and 2 *μ*g/mL of TPCK-trypsin (1 : 1,000 in MEM medium) in each well, and incubated at 37°C again. The cytopathic effect was monitored daily. When 80% of the cells demonstrated pathological changes or the fifth day of the culture was reached, the cells were frozen and thawed two or three times. The supernatant of culture solution was harvested and virus infection was confirmed by RT-PCR.

### 2.5. RT-PCR for Influenza A (H7N9) Virus

The H7N9 RT-PCR detection kit used in our study was from Shanghai Zhijiang Biotechnology Co. Ltd. and Guangzhou Da'an Biotechnology Co. Ltd. RNA was first extracted from specimens according to the manufacturer's instruction. A 25 *μ*L reaction system was set up containing 2 *μ*L template RNA, 1 *μ*L 25× RT-PCR enzyme mix, 0.5 *μ*L Probe (20 *μ*M), 1 *μ*L of each of the primers (10 *μ*M), 12.5 *μ*L of 2x RT-PCR master mixes, and 7 *μ*L RNase free water. The test was performed using a LightCycler 1.2 (Roche, Germany). Amplification conditions were set as follows: 45°C for 5 min, 95°C for 30 s, 40 cycles of 95°C for 5 s, and 60°C for 20 s.

### 2.6. Statistical Analysis

Statistical analysis was performed using SPSS 15.0. Categorical variables were tested using the Chi-squared test, where *P* < 0.05 was considered significant.

## 3. Results

### 3.1. Analytic Sensitivity and Specificity in Testing Recombinant Influenza Hemagglutinin Proteins by GICA

We created and tested a panel consisting of different concentrations (1 ng/mL, 10 ng/mL, 100 ng/mL, and 1000 ng/mL) of recombinant HA proteins of two H7N9 viruses as well as an H7N7 virus. A lower limit of detection (LOD) of 10 ng/mL was obtained (Table 1). No cross-reactions were observed when recombinant HA proteins from H1N1, H3N2, H5N1, H5N9, and H9N2 viruses were tested (Table 1). We also tested the specificity of GICA with virus lysates, including previously isolated H1N1, H9N2, and H5N9 viruses (shown in Table 1) and H1N1, H5N1, and H3N2 viruses isolated from different sources (shown in Supplementary Table 1 available online at http://dx.doi.org/10.1155/2014/425051).

### 3.2. The Positive Rate of H7N9 Virus Detection by Viral Culture, GICA, and RT-PCR When Testing Clinical Samples

From a total of 140 samples from 35 influenza H7N9 patients, 12 samples tested positive for H7N9 virus by viral culture, 34 samples tested positive by GICA, and 99 samples tested positive by RT-PCR. In addition, all throat swabs and sputum specimens from patients with other respiratory pathogen infections tested negative by GICA (data shown in Supplementary Table 2).

### 3.3. Comparison between GICA and RT-PCR

A comparison of the results of RT-PCR and GICA tests is shown in Table 2. Relative to RT-PCR, the sensitivity for GICA was 33.33%, the specificity was 97.56%, the positive predictive value (PPV) was 97.06%, and the negative predictive value (NPV) was 37.74% (Table 2). Further analysis showed that the median Ct value of the RT-PCR assay for all samples was 31.92 (range 16.68–47.09), and 72% of RT-PCR Ct values for positive samples determined by GICA were below 30. The positivity rate from GICA was up to 60% for samples with Ct values below 30 but was only 15.25% for samples with Ct values above 30 (Table 3).

Since multiple samples were taken from some patients infected with H7N9 virus, we analyzed the performance of the GICA with samples collected at different times during the infection. We defined the reported time of contact with poultry by patients as the onset time for H7N9 infection. As shown in Table 4, the positive rate of GICA tests for samples collected in the period from 8 to 21 days after contact with poultry was higher than for samples collected before or after this period. We also found the Ct values for samples collected during this period were lower, indicating higher viral load for these samples. The sensitivity of GICA relative to RT-PCR was also much higher for samples collected in this period.

### 3.4. Comparison between GICA and Viral Culture

A comparison of the results of viral culture and GICA tests is shown in Table 5. Relative to viral culture, the sensitivity of the GICA test was 91.67%, the specificity was 82.03%, the PPV was 32.35%, and the NPV was 99.06% (Table 5). Of the 12 samples that tested positive by viral culture, five were throat swab samples with Ct values from 25.28 to 31.76, and seven were sputum samples with Ct values from 16.68 to 27.20; these values were lower than the median Ct value for all samples.

### 3.5. Comparison of Positive Rates among Different Types of Samples

We further examined the positivity rate for H7N9 viruses among different categories of samples and found that the positivity rate of sputum specimens was higher than those of the other types of specimens, especially for GICA with a positivity rate of 46.43% for sputum specimens, while rates of 13.33% and 5.13% were observed for throat swab and fecal specimens, respectively (Table 6). The median Ct values for sputum, throat swab, and fecal specimens were 28.96 (range, 16.68–38.09), 36.20 (range, 25.28–40.63), and 35.29 (range, 29.89–47.09), respectively, indicating that the viral load of H7N9 in sputum is higher than in the other samples (*P* < 0.01).

## 4. Discussion

Antiviral therapy during the early stages of influenza infection can greatly decrease the incidence of acute respiratory distress syndrome (ARDS) and mortality from influenza virus infections [2]. Early treatment with antiviral therapy requires the early diagnosis of H7N9 infection. However, most of the patients from the current H7N9 epidemic in China were not diagnosed early enough due to the limited technical resources in the primary care settings they visited [14]. RT-PCR is a sensitive and specific assay for the rapid detection of influenza viruses and was widely used for the diagnosis of H7N9 infection. However, this technique was limited to highly equipped hospitals and laboratories.

As a rapid and simple detection method, GICA is widely applied in detecting influenza viruses [11, 15]. In our study, we tested a new GICA for the rapid diagnosis of H7 influenza A viruses and found that the assay specifically reacted with recombinant HA proteins from H7 subtype viruses, including H7N7 and H7N9, but did not react with recombinant HA proteins from other HA subtypes including H1N1, H3N2, and H5N1. These results indicated that the new GICA successfully detects influenza A virus expressing the H7 HA protein, such as H7N9 and H7N7. For tests on clinical samples, as we expected, RT-PCR was the most sensitive assay for the detection of H7N9 viruses compared with viral culture and GICA. However, GICA performed as well as RT-PCR in identifying H7N9-negative samples, regardless of whether the samples were collected from patients infected with H7N9 virus or other respiratory pathogens; that is to say, the specificity of the two tests was comparable. Furthermore, we also found that GICA was more sensitive when testing samples with a high viral load than those with a low viral load. We also found that GICA performed better in samples collected in the period from 8 to 21 days after contact with poultry, which may be related to the higher viral load of the samples in this period. These results indicate that GICA is a sensitive and rapid method for the identification of H7N9-infected patients whose disease may progress more rapidly and have stronger infectivity. Multipoint sampling in the early stage of infection can help to improve the positive rate of diagnosis by GICA. The GICA is more sensitive and specific than viral culture for the detection of H7N9 viruses. The results of GICA are also comparable to the performance of other rapid test kits recommended by the WHO [16, 17]. For example, compared with viral culture, Directigen Flu AqB has been shown to have a sensitivity of 43.8% and specificity of 99.7% [16] and the QuickVue Influenza Test has been shown to have a sensitivity of 70.4% and specificity of 97.7% [17].

We also found that sputum specimens were more likely to test positive for H7N9 viruses than throat swabs or fecal samples, which may be associated with higher viral loads in sputum specimens. Studies have shown that H7N9 viruses colonize the lower respiratory tract [7, 18]. Therefore more H7N9 viruses would be contained in sputum derived from the lower respiratory tract of patients. Interestingly, some fecal specimens from patients with high viral loads tested positive by RT-PCR and GICA, but negative by viral culture. This suggested that there were residual fragments of H7N9 virus in the feces. Further study is needed to determine whether there are whole virus particles in feces and whether there are host cells containing H7N9 virus in the human intestinal tract.

## 5. Conclusion

In conclusion, although the GICA-based test can only detect HA proteins from H7 subtypes, considering the lack of assays for the rapid detection and identification of both H7 and N9 proteins simultaneously that are also suitable for resource-limited primary care settings, GICA remains a reliable, rapid, and convenient method for the screening and preliminary diagnosis of influenza A (H7N9) infection in the early stage, especially from sputum specimens containing a high viral load. However, negative results by GICA are not definitive and other methods should be used for confirmation such as influenza virus RT-PCR in patients with a high clinical suspicion of this infection.

## Supplementary Material

Supplementary Table 1. To determine the specificity of the GICA with different influenza A virus, we tested lysates of several non-H7 influenza A virus by GICA, including H1N1, H5N1and H3N2 virus with different sources. The results were negative for all the tests.
Supplementary Table 2. We also tested the specificity of GICA in 91 throat swabs and sputum samples from patients with other respiratory pathogen infections, including mycobacterium tuberculosis, mycoplasma pneumonia, H1N1 virus and measles virus. All samples tested negative by GICA.

## Figures and Tables

**Table 1:** Analytic sensitivity and specificity in testing recombinant influenza hemagglutinin (HA) proteins by GICA^a^.

Proteins tested	Virus Strains	Concentration (ng/mL)	Results by GICA
Recombinant HA	A/Shanghai/2/2013 (H7N9)	1	+
		10	+
		100	+
		1000	+
Recombinant HA	A/Anhui/1/2013 (H7N9)	1	+
		10	+
		100	+
		1000	+
Recombinant HA	A/Netherlands/2/19/03 (H7N7)	1	−
		10	+
		100	+
		1000	+
Recombinant HA	A/Hubei/1/2010 (H5N1)	1000	−
Recombinant HA	A/Victoria/361/2011 (H3N2)	1000	−
Recombinant HA	A/California/06/2009 (H1N1)	1000	−
Virus lysates	H5N9	1000	−
Virus lysates	H9N2	1000	−
Virus lysates	H1N1	1000	−

^a^GICA: gold immunochromatographic assay.

**Table 2:** Detection of influenza A (H7N9) virus by GICA^a^ and RT-PCR assay.

GICA	RT-PCR		
	Positive(+)	Negative(−)	Total
Positive(+)	33	1	34
Negative(−)	66	40	106
Total	**99**	**41**	**140**

^ b^Sensitivity	Specificity	PPV	NPV

33.33%	97.56%	97.06%	37.74%

^a^GICA: gold immunochromatographic assay.

^b^These parameters for GICA were obtained in reference to the RT-PCR assay.

**Table 3:** Comparison of RT-PCR and GICA^a^ for the detection of H7N9 positive samples.

Ct values for RT-PCR	No. of samples	No. of GICA positive	Positivity rate (%)
≤25	10	8	80.00
25–30	30	16	53.33
30–35	28	8	28.57
>35	31	1	3.23

^a^GICA: gold immunochromatographic assay.

**Table 4:** The performance of GICA and RT-PCR assay with samples collected at different time points of infection.

Days after contact with poultry	Number of samples	RT-PCR assay		GICA		Sensitivit^a^ (%)	Specificity^a^ (%)
		Positive sample number (%)	Ct value median (range)	Positive sample number (%)	Ct value median (range)		
0–7	11	7 (63.64%)	34.42 (22.77–39.16)	2 (18.18%)	29.37 (27.68–31.06)	28.57%	100.00%
8–10	23	17 (73.91%)	31.03 (16.68–37.87)	7 (30.43%)	26.39 (16.68–30.40)	41.18%	100.00%
11–14	45	36 (80.00%)	33.44 (23.10–36.48)	13 (28.89%)	27.72 (24.86–32.51)	36.11%	100.00%
15–21	42	29 (69.05%)	34.72 (19.15–45.08)	11 (26.19%)	29.04 (19.15–30.79)	34.48%	92.31%
>22	19	10 (52.63%)	36.20 (23.48–47.09)	1 (5.26%)	31.17	10.00%	100.00%

^a^These parameters for GICA were obtained in reference to the RT-PCR assay.

**Table 5:** Detection of influenza A (H7N9) virus by GICA^a^ and viral culture.

GICA	Viral culture		
	Positive(+)	Negative(−)	Total
Positive(+)	11	23	34
Negative(−)	1	105	106
Total	**12**	**128**	**140**

^ b^Sensitivity	Specificity	PPV	NPV

91.67%	82.03%	32.35%	99.06%

^a^GICA: gold immunochromatographic assay.

^b^These parameters for GICA were obtained in reference to viral culture.

**Table 6:** The positivity rate of H7N9 virus by GICA^a^, RT-PCR and viral culture among different kinds of samples.

Type of samples	No. of samples	No. of RT-PCR positive (%)	No. of viral culture positive (%)	No. of GICA positive (%)
Sputum	56	55 (98.21)	7 (12.50)	26 (46.43)
Throat swab	45	28 (62.22)	5 (11.11)	6 (13.33)
Feces	39	16 (41.03)	0 (0)	2 (5.13)

^a^GICA: gold immunochromatographic assay.
