# Supplementary material for: Comparison of a New Gold Immunochromatographic Assay for the Rapid Diagnosis of the Novel Influenza A (H7N9) Virus with Cell Culture and a Real-Time Reverse-Transcription PCR Assay
Source: Biomed Res Int. 2014 Apr 14;2014:425051. doi: 10.1155/2014/425051 (PMC4009114; doi:10.1155/2014/425051)
Supplement: Supplementary file 1 — Supplementary Table 1. To determine the specificity of the GICA with different influenza A virus, we tested lysates of several non-H7 influenza A virus by GICA, including H1N1, H5N1and H3N2 virus with different sources. The results were negative for all the tests. Supplementary Table 2. We also tested the specificity of GICA in 91 throat swabs and sputum samples from patients with other respiratory pathogen infections, including mycobacterium tuberculosis, mycoplasma pneumonia, H1N1 virus and measles virus. All samples tested negative by GICA. [file 425051.f1.doc]

**Supplementary** Table 1. Quality control panel tested by GICA

| Code No. | Virus Strains | Subtypes | HA Titers | Results by GICA* |
| --- | --- | --- | --- | --- |
| N1 | California/7/2009 | H1N1 | 32 | － |
| N2 | Perth/16/2009 | H3N2 | 128 | － |
| N3 | Chongqin Yuzhong/1_50_/2007 | H1N1 | 32 | － |
| N4 | A/Fujian Tongan/196/2009 | H5N1 | 128 | － |
| N5 | A/Brisban/10/2007 | H3N2 | 128 | － |
| N6 | A/Hubei Jiangan/1139/2009 | H3N2 | 32 | － |
| N7 | A/Yunnan/1145/2005 | H3N2 | 128 | － |
| N8 | A/Hubei Beihu/1143/2011 | H3N2 | 64 | － |
| N9 | A/Liaoning Huanggu/1183/2007 | H1N1 | 256 | － |
| N10 | A/Jiangxi Donghu/312/2006 | H3N2 | 128 | － |
| N11 | A/Anhui Baohe/137/2008 | H3N2 | 64 | － |
| N12 | A/Guangdong Nongan/SWL112/2010 | H1N1 | 128 | － |

* Each sample was tested 2-3 times.

| **Supplementary table 2. Clinical samples from patients with non H7N9 influenza A virus and other respiratory pathogens** | | | | | | |
| --- | --- | --- | --- | --- | --- | --- |
| **No.** | **Gender** | **Age** | **Diagnosis** | **Pathogen** | **Sample type** | **GICA test** |
| 1 | male | 79 | Pulmonary tuberculosis | Mycobacterium tuberculosis | sputum | negative |
| 2 | male | 67 | Pulmonary tuberculosis | Mycobacterium tuberculosis | sputum | negative |
| 3 | male | 75 | Pulmonary tuberculosis | Mycobacterium tuberculosis | sputum | negative |
| 4 | male | 66 | Pulmonary tuberculosis | Mycobacterium tuberculosis | sputum | negative |
| 5 | male | 75 | Pulmonary tuberculosis | Mycobacterium tuberculosis | sputum | negative |
| 6 | male | 38 | Pulmonary tuberculosis | Mycobacterium tuberculosis | sputum | negative |
| 7 | male | 80 | Pulmonary tuberculosis | Mycobacterium tuberculosis | sputum | negative |
| 8 | female | 63 | Pulmonary tuberculosis | Mycobacterium tuberculosis | sputum | negative |
| 9 | female | 59 | Pulmonary tuberculosis | Mycobacterium tuberculosis | sputum | negative |
| 10 | male | 63 | Pulmonary tuberculosis | Mycobacterium tuberculosis | sputum | negative |
| 11 | male | 75 | pneumoniae | Mycoplasma pneumoniae | sputum | negative |
| 12 | female | 62 | pneumoniae | Mycoplasma pneumoniae | sputum | negative |
| 13 | female | 67 | pneumoniae | Mycoplasma pneumoniae | sputum | negative |
| 14 | female | 73 | pneumoniae | Mycoplasma pneumoniae | sputum | negative |
| 15 | male | 75 | pneumoniae | Mycoplasma pneumoniae | sputum | negative |
| 16 | male | 41 | pneumoniae | Mycoplasma pneumoniae | sputum | negative |
| 17 | female | 62 | pneumoniae | Mycoplasma pneumoniae | sputum | negative |
| 18 | male | 75 | pneumoniae | Mycoplasma pneumoniae | sputum | negative |
| 19 | female | 73 | pneumoniae | Mycoplasma pneumoniae | sputum | negative |
| 20 | male | 66 | pneumoniae | Mycoplasma pneumoniae | sputum | negative |
| 21 | male | 75 | pneumoniae | Mycoplasma pneumoniae | sputum | negative |
| 22 | male | 70 | pneumoniae | Mycoplasma pneumoniae | sputum | negative |
| 23 | male | 30 | pneumoniae | Mycoplasma pneumoniae | sputum | negative |
| 24 | male | 75 | pneumoniae | Mycoplasma pneumoniae | sputum | negative |
| 25 | male | 41 | pneumoniae | Mycoplasma pneumoniae | sputum | negative |
| 26 | male | 77 | Fever and respiratory syndrome with unknown origin | influenza A negative | throat swab | negative |
| 27 | female | 46 | Fever and respiratory syndrome with unknown origin | influenza A negative | throat swab | negative |
| 28 | female | 28 | Fever and respiratory syndrome with unknown origin | influenza A negative | throat swab | negative |
| 29 | male | 69 | Fever and respiratory syndrome with unknown origin | influenza A negative | throat swab | negative |
| 30 | female | 49 | Fever and respiratory syndrome with unknown origin | influenza A negative | throat swab | negative |
| 31 | female | 79 | Fever and respiratory syndrome with unknown origin | influenza A negative | throat swab | negative |
| 32 | male | 20 | Fever and respiratory syndrome with unknown origin | influenza A negative | throat swab | negative |
| 33 | female | 59 | Fever and respiratory syndrome with unknown origin | influenza A negative | throat swab | negative |
| 34 | male | 29 | Fever and respiratory syndrome with unknown origin | influenza A negative | throat swab | negative |
| 35 | male | 59 | Fever and respiratory syndrome with unknown origin | influenza A negative | throat swab | negative |
| 36 | female | 23 | Fever and respiratory syndrome with unknown origin | influenza A negative | throat swab | negative |
| 37 | female | 27 | Fever and respiratory syndrome with unknown origin | influenza A negative | throat swab | negative |
| 38 | male | 23 | Fever and respiratory syndrome with unknown origin | influenza A negative | throat swab | negative |
| 39 | male | 37 | Fever and respiratory syndrome with unknown origin | influenza A negative | throat swab | negative |
| 40 | male | 45 | Fever and respiratory syndrome with unknown origin | influenza A negative | throat swab | negative |
| 41 | female | 63 | Fever and respiratory syndrome with unknown origin | influenza A negative | throat swab | negative |
| 42 | male | 60 | Fever and respiratory syndrome with unknown origin | influenza A negative | throat swab | negative |
| 43 | female | 79 | Fever and respiratory syndrome with unknown origin | influenza A negative | throat swab | negative |
| 44 | male | 26 | Fever and respiratory syndrome with unknown origin | influenza A negative | throat swab | negative |
| 45 | female | 57 | Fever and respiratory syndrome with unknown origin | influenza A negative | throat swab | negative |
| 46 | female | 86 | Fever and respiratory syndrome with unknown origin | influenza A negative | throat swab | negative |
| 47 | male | 81 | Fever and respiratory syndrome with unknown origin | influenza A negative | throat swab | negative |
| 48 | male | 27 | Fever and respiratory syndrome with unknown origin | influenza A negative | throat swab | negative |
| 49 | male | 60 | Fever and respiratory syndrome with unknown origin | influenza A negative | throat swab | negative |
| 50 | male | 84 | Fever and respiratory syndrome with unknown origin | influenza A negative | throat swab | negative |
| 51 | female | 25 | Fever and respiratory syndrome with unknown origin | influenza A negative | throat swab | negative |
| 52 | male | 50 | Fever and respiratory syndrome with unknown origin | influenza A negative | throat swab | negative |
| 53 | female | 24 | Fever and respiratory syndrome with unknown origin | influenza A negative | throat swab | negative |
| 54 | female | 39 | Fever and respiratory syndrome with unknown origin | influenza A negative | throat swab | negative |
| 55 | female | 22 | Fever and respiratory syndrome with unknown origin | influenza A negative | throat swab | negative |
| 56 | female | 17 | Fever and respiratory syndrome with unknown origin | influenza A negative | throat swab | negative |
| 57 | male | 57 | Fever and respiratory syndrome with unknown origin | influenza A negative | throat swab | negative |
| 58 | female | 47 | Fever and respiratory syndrome with unknown origin | influenza A negative | throat swab | negative |
| 59 | female | 65 | Fever and respiratory syndrome with unknown origin | influenza A negative | throat swab | negative |
| 60 | female | 40 | Fever and respiratory syndrome with unknown origin | influenza A negative | throat swab | negative |
| 61 | male | 70 | Fever and respiratory syndrome with unknown origin | influenza A negative | throat swab | negative |
| 62 | female | 59 | Fever and respiratory syndrome with unknown origin | influenza A negative | throat swab | negative |
| 63 | female | 59 | Fever and respiratory syndrome with unknown origin | influenza A negative | throat swab | negative |
| 64 | female | 71 | Fever and respiratory syndrome with unknown origin | influenza A negative | throat swab | negative |
| 65 | male | 42 | Fever and respiratory syndrome with unknown origin | influenza A negative | throat swab | negative |
| 66 | female | 60 | Fever and respiratory syndrome with unknown origin | influenza A negative | throat swab | negative |
| 67 | female | 33 | Fever and respiratory syndrome with unknown origin | influenza A negative | throat swab | negative |
| 68 | female | 65 | Fever and respiratory syndrome with unknown origin | influenza A negative | throat swab | negative |
| 69 | male | 49 | Fever and respiratory syndrome with unknown origin | influenza A negative | throat swab | negative |
| 70 | female | 60 | Fever and respiratory syndrome with unknown origin | influenza A negative | throat swab | negative |
| 71 | female | 35 | Fever and respiratory syndrome with unknown origin | influenza A negative | throat swab | negative |
| 72 | female | 61 | Fever and respiratory syndrome with unknown origin | influenza A negative | throat swab | negative |
| 73 | male | 54 | Fever and respiratory syndrome with unknown origin | influenza A negative | throat swab | negative |
| 74 | male | 75 | influenza A | H7N9 negative | throat swab | negative |
| 75 | male | 66 | influenza A | H7N9 negative | throat swab | negative |
| 76 | female | 37 | influenza A | H7N9 negative | throat swab | negative |
| 77 | female | 67 | influenza A | H7N9 negative | throat swab | negative |
| 78 | male | 75 | influenza A | H7N9 negative | throat swab | negative |
| 79 | male | 66 | Measles | Measles virus | throat swab | negative |
| 80 | male | 86 | Measles | Measles virus | throat swab | negative |
| 81 | male | 66 | Measles | Measles virus | throat swab | negative |
| 82 | male | 63 | Measles | Measles virus | throat swab | negative |
| 83 | male | 30 | Measles | Measles virus | throat swab | negative |
| 84 | male | 66 | Measles | Measles virus | throat swab | negative |
| 85 | female | 67 | influenza A | H1N1 | throat swab | negative |
| 86 | male | 75 | influenza A | H1N1 | throat swab | negative |
| 87 | male | 86 | influenza A | H1N1 | throat swab | negative |
| 88 | male | 67 | influenza A | H1N1 | throat swab | negative |
| 89 | male | 30 | influenza A | H1N1 | throat swab | negative |
| 90 | male | 43 | influenza A | H1N1 | throat swab | negative |
| 91 | female | 62 | influenza A | H1N1 | throat swab | negative |
